# Colour as a backup for scent in the presence of olfactory noise: testing the efficacy backup hypothesis using bumblebees (*Bombus terrestris*)

**DOI:** 10.1098/rsos.170996

**Published:** 2017-11-29

**Authors:** David A. Lawson, Heather M. Whitney, Sean A. Rands

**Affiliations:** School of Biological Sciences, University of Bristol, Bristol BS8 1TQ, UK

**Keywords:** floral displays, multimodality, signalling, pollination, floral scent

## Abstract

The majority of floral displays simultaneously broadcast signals from multiple sensory modalities, but these multimodal displays come at both a metabolic cost and an increased conspicuousness to floral antagonists. Why then do plants invest in these costly multimodal displays? The efficacy backup hypothesis suggests that individual signal components act as a backup for others in the presence of environmental variability. Here, we test the efficacy backup hypothesis by investigating the ability of bumblebees to differentiate between sets of artificial flowers in the presence of either chemical interference or high wind speeds, both of which have the potential to impede the transmission of olfactory signals. We found that both chemical interference and high wind speeds negatively affected forager learning times, but these effects were mitigated in the presence of a visual signal component. Our results suggest that visual signals can act as a backup for olfactory signals in the presence of chemical interference and high wind speeds, and support the efficacy backup hypothesis as an explanation for the evolution of multimodal floral displays.

## 1. Background

Floral displays often consist of multiple signal components which transmit information simultaneously across multiple sensory modalities. These displays are complex in that they broadcast visual, olfactory, tactile, gustatory, electrostatic and even acoustic information [1–3]. These signals are metabolically costly and difficult to produce [4], and additional floral display components add not only the metabolic cost of the display component itself [5–7] but also the risk of attracting herbivores [8] and predators which target pollinating insects [9]. So, why invest in these multimodal displays?

Many non-exclusive hypotheses have been developed to explain the ubiquity of multimodal or multicomponent signals in nature [10–13], including frameworks dedicated solely to floral signal complexity [14]. These hypotheses are frequently categorized as relating to either the content of a signal being transmitted, the efficacy of a signal (and how effectively the signal is transmitted through the environment), or the influence of one signal on the receiver's response to another [13]. Several of the efficacy-based hypotheses relate to the transmission of signals in varying environmental conditions, which are known to affect the generation, reception and transmission of signals [15]. Varying levels of light, humidity, wind speed and wind direction all have the potential to reduce the efficacy of a signal. The efficacy backup hypothesis [13] states that individual signals act as a backup to others in varying environmental conditions. By communicating signals across multiple sensory modalities, signal producers increase the likelihood of a signal being received. An example of this is seen in wolf spiders (*Schizocosa ocreata*) where males, which use both visual and vibratory signals during courtship displays, were observed using more visual signals while on substrates which inhibit the transmission of seismic signals [16]. This ‘backing up' of signals is also seen in plant–pollinator systems where floral scents act as a backup to floral colour signals in low-light conditions in bumblebees *Bombus impatiens* [17]. Floral displays present a unique opportunity to investigate this hypothesis as plant–pollinator relationships have evolved in the presence of multiple abiotic and biotic environmental conditions which have the potential to obscure the transmission of signals.

Plants produce a range of volatile organic compounds (VOCs), which mediate interactions between plants and their pollinators, seed dispersers and potential antagonists [8,18]. These VOCs primarily move through the environment through two processes: diffusion, whereby molecules move from a region of high concentration to a region of low concentration; and advection, whereby molecules are transported through the motion of a moving fluid [19,20]. It is also likely that convection [21] and boundary layer effects [22] are also affecting this movement of VOC molecules. Advection in particular is important in the development of odour plumes, which are high concentrations of VOCs interspersed with air [20,23]. As VOCs cannot be directed and potentially move slowly through the environment (when compared with signals of other modalities, such as visual information), there is more time for environmental noise to affect their transmission. This, coupled with the fact that the vastly different physiologies of plants and their pollinators are potentially subjected to a greater array of environmental interference compared with more closely related signallers and receivers, puts volatile transmission at particular risk to environmental noise [24].

VOCs can be affected by environmental noise during their production by the plant, or during their transfer through the air, or during their reception and processing by the receiver [24]. Here, we examine two types of environmental noise which affect the transmission of VOCs during their transfer through the air: chemical interference and air turbulence. These have been proposed as the two main sources of noise affecting the transmission of floral VOCs, and have the potential to affect both location and context information [24–26].

Chemical interference occurs if different plant species share VOCs making the desired flowers more difficult to localize [25], or when fragrance blends from multiple species with varying VOC ratios mix in such a way as to change the content of a signal to a receiver [24]. Considering that over 1700 VOCs have been identified from plant species, and more importantly the high frequency of particular single compounds such as the monoterpenes limonene, myrcene and linalool in floral scents [27], there is substantial potential for these interactions. Airborne chemicals, such as tropospheric ozone, hydroxyl radicals and nitrate radicals, can also interact with VOCs [28,29].

Air turbulence, which is defined as random fluctuations in the velocity of a fluid [30] and is highly affected by wind, also has the potential to affect VOC transmission through the disruption of odour plumes [20,23]. Low-to-medium wind speeds are beneficial to flying insects as they can narrow an odour plume into a straighter structure which can be more easily followed, although some turbulence-generated heterogeneity in the plume may be important to allow orientation [31]. However, wind speed becomes detrimental above a certain threshold, as increased turbulence disrupts the structure of odour plumes increasing their spatial and temporal heterogeneity [19,30,32–36]. This connection is implied in male long-horned bees, where search times are negatively correlated with wind speed for winds between 0 and 2.7 m s^−1^, but positively correlated at wind speeds above 2.7 m s^−1^ [37]. These increases to search times as well as limited flight movement control during greater wind speeds [38] highlight the detrimental effect high wind speeds can have on pollination interactions.

We argue here that bumblebees have the capacity to use visual information as a backup for olfactory information in the presence of environmental noise. Previous tests of the efficacy backup hypothesis have shown bumblebees to use scents to back up colour signals in low-light conditions [17], giving precedence for sensory backup of other modalities. This, coupled with both the susceptibility of olfactory signal transfer to noise [24] and the fact that scent rather than colour has been shown to be the preferred discriminating factor in honeybees [39], suggests that this sensory backup could take place. In this study, we test the converse possibility of Kaczorowski *et al*. [17], who suggest that under well-lit conditions, visual signals can play an efficacy backup role for olfactory signals when the latter are obfuscated by noise. We used essential oils from other plant species to simulate chemical interference (hereafter referred to as the chemical interference test), and an electric fan to simulate high wind speeds (hereafter called the wind-simulation test). We recorded learning times, the number of successful drinks and the number of correct choices a forager bee made after landing on an artificial flower. We hypothesize that both chemical interference and interference from high wind speeds will detrimentally affect bee foraging efficiency and that these effects will be mitigated with the introduction of an additional visual signal component to act as a backup.

## 2. Material and methods

### 2.1. Flight arena and bumblebee colony conditions

All three experiment types were carried out in wooden framed 72 × 104 × 30 cm flight arenas topped with UV-transparent Perspex with the floor covered in Advance Green gaffer tape (Stage Electric, UK). Flight arenas were connected to the plastic nesting box of flower naive *Bombus terrestris dalmatinus* colonies (Biobest, Sustainable Crop Management, Belgium) via a transparent gated tube which could be manually manipulated to regulate which bees, and how many, could enter or leave the flight arena. Forty-six Sylvania Activa 172 Professional 36 W fluorescent tubes (Germany) on a 12 L : 12 D regime were used to simulate natural illumination. Bees were fed 30% sucrose solution daily after experiments had taken place and pollen was added directly to the colony three days a week. A total of ten colonies were used over the three experiment types (summarized in the electronic supplementary material, tables S1 and S2)—in analysis, we assumed that there was sufficient behavioural difference between individuals to ignore any colony effects, as is discussed in Thomson & Chittka [40]. Foraging individuals were marked on the thorax with non-toxic paint in order to be identified and used during the experiments.

### 2.2. Artificial flowers

**Figure 1.**
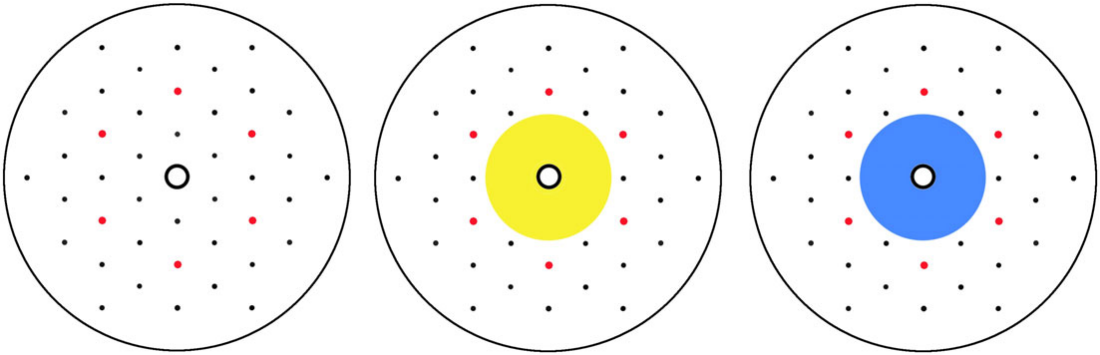
Artificial flower discs used within the experiments. Red points are used to display where scent solutions are placed. The first disc has no visual component, whereas the second disc has a yellow visual component and the third disc a blue visual component. Artificial flowers can be scented with either diluted peppermint or lavender solutions.

Ten white Perspex discs (80 mm diameter, 3 mm width) were used as foraging stimuli (artificial flowers) during each experiment. Each disc had 43 holes (2 mm diameter) in a hexagonal pattern (figure 1), with a transparent plastic cover placed on top of each disc. Each cover had 2 mm holes corresponding to those on the disc and an upturned 0.5 ml Eppendorf container lid glued to the centre of the disc surface to be used to contain a sucrose reward or water. The plastic covers were used in order for the discs to be cleaned thoroughly after experiments without compromising the Eppendorf feeding wells. At the beginning of each experiment, self-adhesive film was placed on the underside of each disc so the holes could contain small amounts of liquid. At the end of each day, this film was removed and the discs were soaked overnight in a detergent solution to remove volatiles and glue.

Within treatments which incorporated scented flowers, five of the ten artificial flowers had a lavender oil solution added using a pipette (1 : 10 mix of lavender essential oil : mineral oil) in a hexagonal arrangement (2.5 µl of oil added to 6 of the 43 holes, figure 1) and in the remaining five artificial flowers a peppermint oil solution was added using a pipette with the same arrangement and amount (1 : 10 mix of peppermint oil : mineral oil. Lavender and peppermint oils were supplied by Amphora Aromatics, Bristol, UK). Within treatments which incorporated visual cues five of the ten artificial flowers had a yellow-coloured disc (Hue: 58; Sat: 80; Lum: 100) and the other five received blue-coloured discs (Hue: 219; Sat: 72; Lum: 100) placed underneath the transparent plastic films on top of each artificial flower. The yellow and blue colours were used as bees are known to perceive and differentiate between these ‘dissimilar' colours [41,42]. Each of these coloured discs was covered on both sides in self-adhesive film for the easy removal of floral volatiles at the end of each experiment. Scented and visual sets were matched so that there were only two different artificial flower groups presented to each forager, e.g. only blue-lavender-scented flowers and yellow-peppermint-scented flowers were presented to one forager and only yellow-lavender-scented flowers and blue-peppermint-scented flowers were presented to another forager.

### 2.3. Flight arena preparation

In each experiment, the flight arena was cleared of bees and the gated tube connected to the nest was blocked. Ten artificial flowers were placed in the flight arena, five from each scent groups (lavender- or peppermint-scented flowers) with a 30% sucrose solution (20 µl) reward placed in the central wells of each disc of one group, and into the central well of the other group 20 µl of distilled water was added as an unrewarding stimulus. Each disc was placed on top of an upturned plastic container (6 cm height, 150 ml, Sterilin UK) and distributed randomly throughout the flight arena.

### 2.4. Experiment 1: chemical interference experiment

Foragers were randomly allocated to one of four groups (18 bees to each group, summarized in the electronic supplementary material, table S1): (A) with no chemical interference and unimodal-scented flowers (control treatment), used to gain a baseline level of foraging efficiency when using olfactory cues in an interference-free environment; (B) with chemical interference and unimodal-scented flowers, used to see the effects of the introduced interference; (C) with chemical interference and bimodal scented and visual flowers, used to see if effects caused by interference (as demonstrated by group B) could be mitigated with the addition of a visual cue; and (D) with chemical interference and unimodal visual flowers, used to demonstrate if there are effects to foraging efficiency when purely visual artificial flowers are presented alongside chemical interference. The three groups with chemical interference (groups B, C and D), which was used to reduce the reliability of recognition cues, had four sets of two upturned Eppendorf lids, each set with 200 µl of one of four essential oils distributed throughout the flight arena. The scents used were essential oils from geranium *Pelargonium graveolens*, bog myrtle *Myrica gale*, juniperberry *Juniperus communis* and camomile Roman *Anthemis nobilis* (from Amphora Aromatics, Bristol, UK), which were applied using a pipette. Group A had no additional scents in the flight arena. This wide range of essential oils, rather than comparatively simpler single odorants, was used as both floral recognition cues and chemical interference to better simulate the complexity of odours which pollinators are presented with in the wild.

Individual marked bees which were naive to both visual and olfactory stimuli, but had experienced drinking from Eppendorf lid wells, were then allowed entry into the flight arena. The sequence of lands on rewarding or non-rewarding artificial flowers was recorded as well as whether the forager drank after landing or abandoned the flower before drinking. Visits to the same flower without visiting another flower in between were not recorded. Flowers which had been visited had their sucrose or water refilled and after each foraging bout the artificial flowers were removed and wiped with ethanol to remove visual cues and foraging pheromones [43]. After this, the artificial flowers were placed back in the flight arena in a different arrangement to avoid foragers learning the location of rewarding artificial flowers.

#### 2.4.1. Behavioural metrics and learning criterion

Foragers were assumed to have satisfactorily learned to discriminate between the two flower groups when eight of the last ten drinks were from rewarding flowers, not counting visits which did not lead to drinks. Only visits which led to drinks were included in this count as it was clearer in these instances that bees made correct choices (positive drinks) or incorrect choices (unrewarding drinks); this was less clear in visits which did not lead to drinks. However, as fine-tuned discrimination of flower odours is known to occur post-landing, and as these visits have implications in terms of benefits to the plant [44,45], we investigated post-landing decisions in our ‘number of correct choices made after landing' comparisons. Forty flower visits were recorded before wiping the artificial flowers with ethanol and focusing on another forager. If the bee had not reached the learning criterion within 40 flower visits, a learning time of 40 was used: this occurred with four bees out of the 72 tested.

### 2.5. Experiment 2: wind simulation experiment

**Figure 2.**
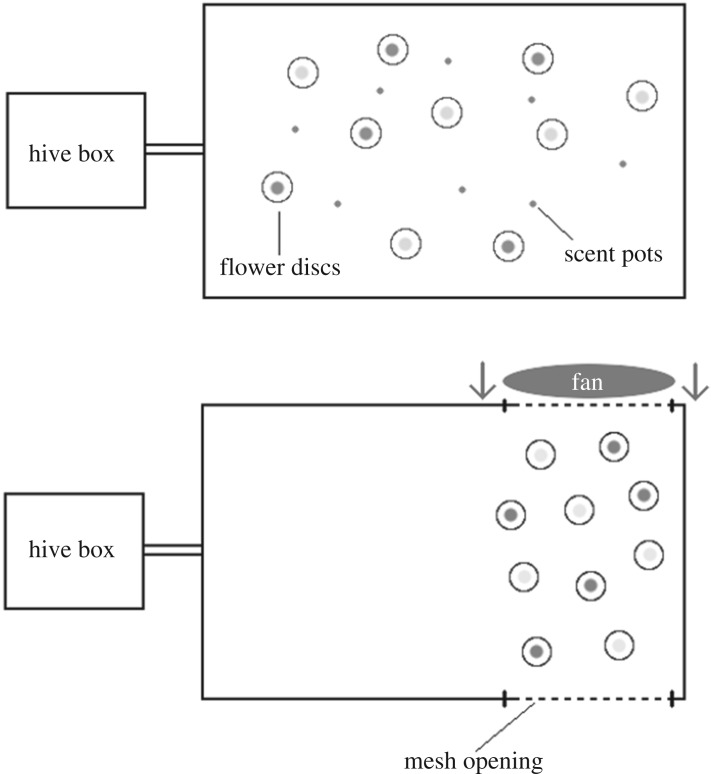
Diagram showing the experimental setup of the two experiments within this study. The first setup displays a typical distribution of artificial flowers and scent pots during the chemical interference experiment, whereas the second setup displays a typical distribution of artificial flowers used during the wind simulation experiment. No comparisons are made between the results of the two experiment types.

Foragers were randomly allocated to one of four groups (18 bees to each group, summarized in electronic supplementary material, table S2) with either: (A) no air movement and unimodal-scented flowers (control treatment); (B) air movement and unimodal-scented flowers; (C) air movement and bimodal scented and visual flowers; or (D) air movement and unimodal visual flowers. These treatment groups were used for the same comparative purposes as those used in experiment 1. All groups had two opposite slats of the flight arena removed and replaced with a metallic mesh held in place with gaffer tape. Groups B, C and D had a fan (Beldray 6 Inch Desk Fan, 15 W power, 15 cm blade diameter, input 220–240 V, 50 Hz) placed on the outside of one of the mesh openings facing into the flight arena creating a corridor where air can pass into the flight arena, pass over the artificial flowers and out of the flight arena from the far opening (figure 2). This new disc arrangement (figure 2), which differs from the arrangement in experiment 1, was chosen to allow air to pass over all artificial flowers. This new arrangement had the potential to affect the foraging behaviour of the bees; therefore, results from experiments 1 and 2 were not directly compared. One group would receive a yellow-coloured disc and the other would receive a blue-coloured disc (same artificial flowers as previous experiment).

At the beginning of the experiment, the fan was turned onto its highest setting with wind speed measured at mean ± s.d. = 1.07 ± 0.86 m s^−1^ (using a Kestrel 4500 pocket weather tracker), which passes the threshold at which odour source finding is compromised in tsetse flies *Glossina pallidipes* [35]. Individual marked forager bumblebees were then allowed entry into the flight arena with the same procedure as the first experiment and an identical learning criterion. Forty flower visits were recorded before wiping the artificial flowers with ethanol and focusing on another forager. If the bee had not reached the learning criterion by the fortieth land the experiment would continue until it met the learning criterion (this occurred with six bees out of a tested 72). Trials which incorporated chemical interference were not performed on the same day as those without chemical interference in order to prevent odour effects on later experiments. For the same reason, all flight arena slats were opened and the flight arena was cleaned after trials which incorporated chemical interference.

### 2.6. Scent preference tests

Scent preference tests were also undertaken to investigate if naive bees had an innate preference to peppermint or lavender. Within these preference tests naive forager bees were presented with 10 artificial flowers (colourless artificial flowers shown in figure 1), five with diluted peppermint oil and five with diluted lavender oil (1 : 10 mix of essential oil : mineral oil) and the first 20 flower visits were recorded. Flowers which had been visited by foragers had their sucrose or water refilled during experiments. Between foraging bouts, artificial flowers were removed and wiped with ethanol.

### 2.7. Analysis

The number of flower visits taken to reach the learning criterion, the number of correct choices after landing and the number of drinks from rewarding flowers between the treatments and control were compared using Kruskal–Wallis tests as the data did not fit the requirements for parametric testing, except for the total number of drinks from rewarding flowers where an analysis of variance was used. *Post hoc* comparisons were conducted using Dunn's tests with Holm–Bonferroni corrections to avoid familywise errors, and a pairwise *t*-test was used for the previously mentioned exception. In order to see if the rewarding scent or colour used had an effect on learning time, we compared the number of flower visits taken to reach the learning criterion between foragers presented with rewarding lavender- and rewarding mint-scented flowers, as well as rewarding yellow flowers and rewarding blue flowers, using Mann–Whitney *U* tests. Scent preference comparisons were undertaken using a one sample *t*-test.

## 3. Results

### 3.1. Experiment 1: chemical interference

#### 3.1.1. Flower visits to reach learning criterion

**Figure 3.**
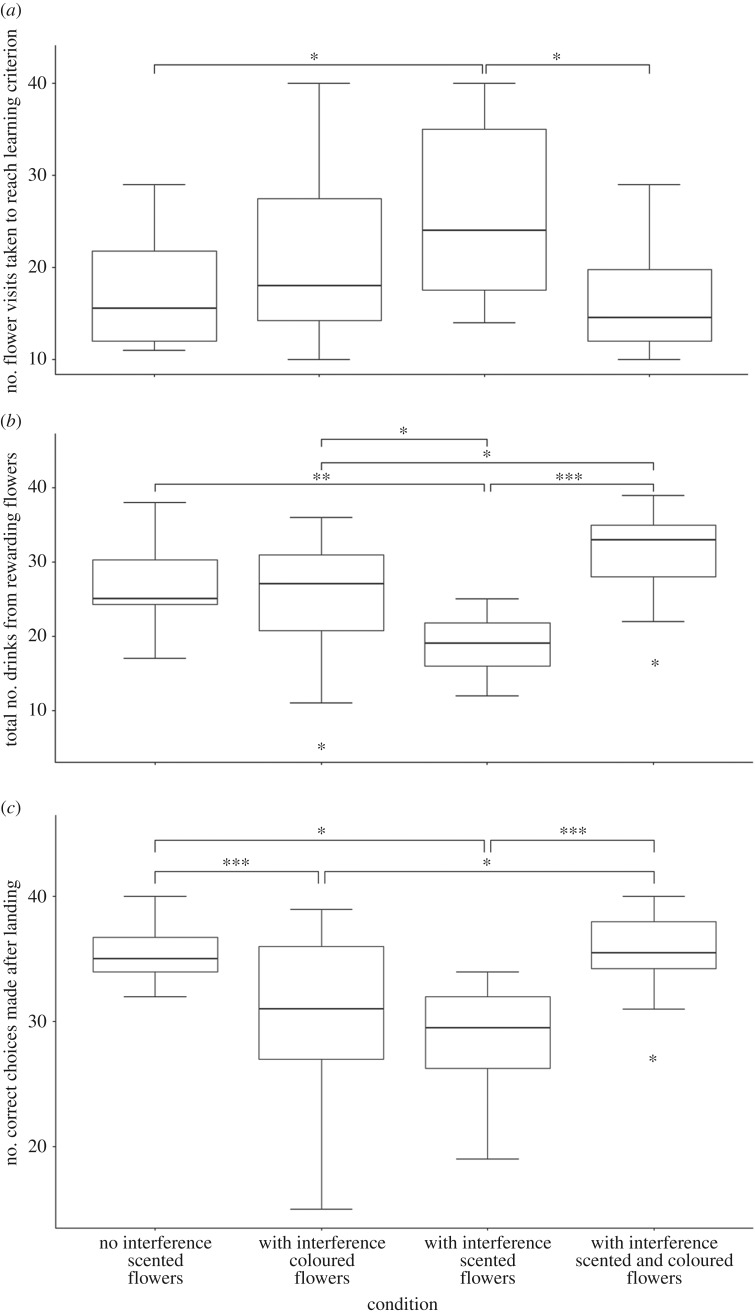
Boxplots showing the foraging and learning behaviour of forager bumblebees under the four treatments considered with chemical interference (Experiment 1). (*a*) The number of flower visits for forager bees to reach the learning criterion of 8 out of 10 consecutive drinks from rewarding flowers, (*b*) the total number of drinks from rewarding flowers and (*c*) the number of correct choices made after landing on a flower; *p ≤ 0.05, **p ≤ 0.01, ***p ≤ 0.001.

The number of flower visits taken to reach the learning criterion was different between the treatments and control (Kruskal–Wallis test: *χ*^2^_3_ = 14.27, *p* = 0.003, figure 3*a*). *Post hoc* comparisons revealed that foragers presented with chemical interference and unimodal scented flowers took more flower visits to reach the learning criterion compared with foragers presented with no chemical interference and unimodal scented flowers (*p* = 0.0117) and those with chemical interference and bimodal scented and coloured flowers (*p* = 0.0015). There was no difference in the number of flower visits taken to reach the learning criterion between the rewarding scents (lavender: learning time = 18.07 ± 7.96 flower visits (mean ± s.d.), peppermint: learning time = 21.44 ± 8.75 flower visits, *N_1_* = 27_,_ *N_2_* = 27_,_ *U* = 267.5, *p* = 0.094) or rewarding colours (blue: learning time = 16.95 ± 7.65 flower visits, yellow: learning time = 21.00 ± 8.35 flower visits, *N_1_* = 19_,_ *N_2_* = 17*, U* = 222, *p* = 0.057).

#### 3.1.2. Total number of drinks from rewarding flowers

The total number of drinks from rewarding flowers was different between the treatments and control (analysis of variance: *F*_3, 68_ = 12.16, *p* < 0.0001, figure 3*b*). *Post hoc* comparisons revealed that foragers presented with chemical interference and unimodal scented flowers drank from fewer rewarding flowers compared with foragers presented with no chemical interference and unimodal scented flowers (*p* = 0.0016), those presented with chemical interference and unimodal coloured flowers (*p* = 0.0136) and those with chemical interference and bimodal scented and coloured flowers (*p* < 0.0001). Foragers presented with chemical interference and unimodal coloured flowers also drank from fewer rewarding flowers compared with those presented with chemical interference and bimodal scented and coloured flowers (*p* = 0.014).

#### 3.1.3. Correct choices made after landing

The number of correct choices made after landing was different between the treatments and control (Kruskal–Wallis test: *χ*^2^_3_ = 27.06, *p* < 0.001, figure 3*c*). *Post hoc* comparisons revealed that foragers presented with both chemical interference and unimodal scented flowers and those presented with chemical interference and unimodal coloured flowers made fewer correct choices after landing compared with foragers presented with no chemical interference and unimodal scented flowers (*p* = 0.001, *p* = 0.032, respectively) and those with chemical interference and bimodal scented and coloured flowers (*p* < 0.0001, *p* = 0.015, respectively).

### 3.2. Experiment 2: air movement

#### 3.2.1. Flower visits to reach learning criterion

**Figure 4.**
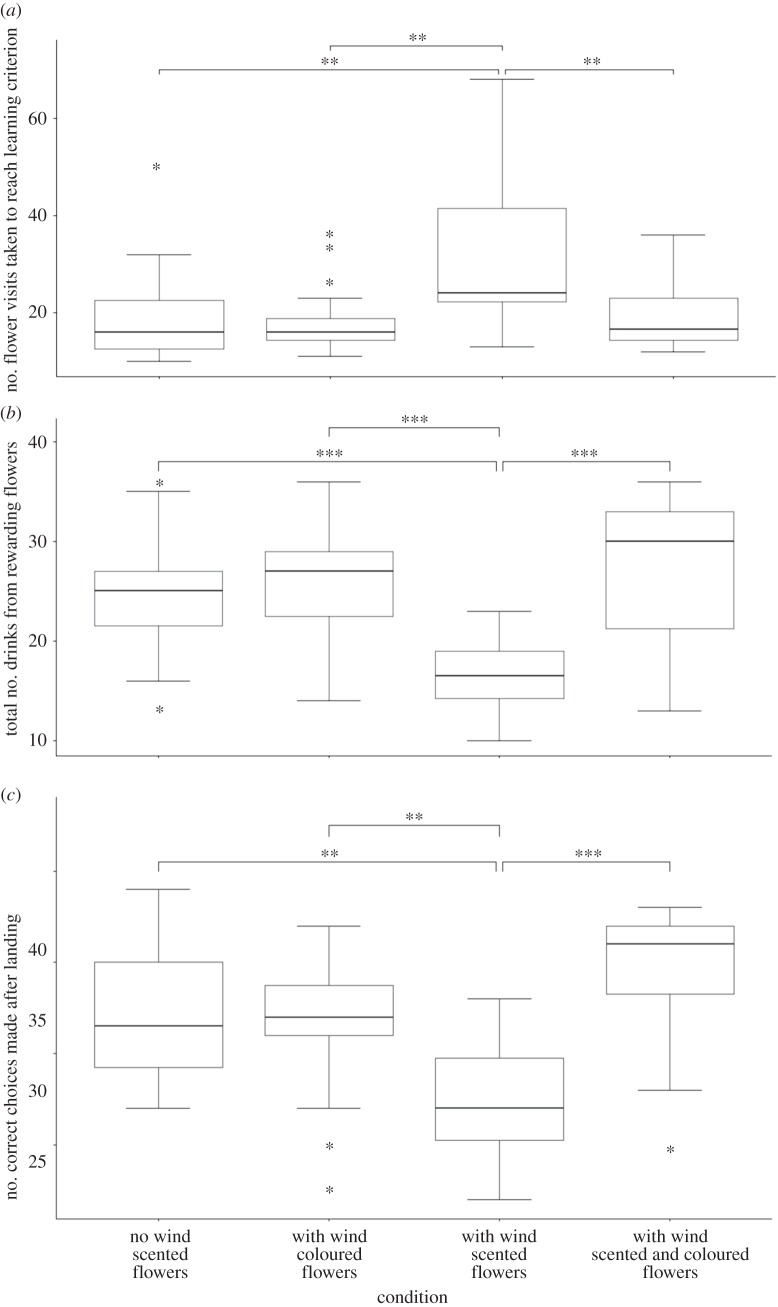
Boxplots showing the foraging and learning behaviour of forager bumblebees under the four treatments with wind disturbance (Experiment 2). (*a*) The number of flower visits for forager bees to reach the learning criterion of 8 out of 10 consecutive drinks from rewarding flowers, (*b*) the total number of drinks from rewarding flowers and (*c*) the number of correct choices made after landing on a flower; *p ≤ 0.05, **p ≤ 0.01, ***p ≤ 0.001.

The number of flower visits taken to reach the learning criterion was different between the treatments and control (Kruskal–Wallis test: *χ*^2^_3_ = 14.3, *p* = 0.003, figure 4*a*). *Post hoc* comparisons revealed that foragers presented with wind and unimodal scented flowers took significantly more flower visits to reach the learning criterion compared with foragers presented with no wind simulation and unimodal scented flowers (*p* = 0.004), those presented with wind simulation and bimodal scented and coloured flowers (*p* = 0.007) and those with wind simulation and unimodal coloured flowers (*p* = 0.005). There was no difference in the number of flower visits taken to reach the learning criterion between the rewarding scents (lavender: learning time = 24.82 ± 14.44 flower visits, peppermint: learning time = 21.62 ± 9.79 flower visits, *N_1_* = 27_,_ *N_2_* = 27*, U* = 413, *p* = 0.406) or rewarding colours (blue: learning time = 17.57 ± 5.96 flower visits, yellow: learning time = 19.87 ± 8.25 flower visits, *N_1_* = 21_,_ *N_2_* = 15*, U* = 172.5, *p* = 0.640).

#### 3.2.2. Total number of drinks from rewarding flowers

The total number of drinks from rewarding flowers was different between the treatments and control (*χ*^2^_3_ = 30.6, *p* < 0.001, figure 4*b*). *Post hoc* comparisons demonstrated that forager bees exposed to wind simulation and unimodal scented flowers made fewer consecutive rewarding drinks compared with foragers presented with no wind simulation and unimodal scented flowers (*p* = 0.001), those presented with wind simulation bimodal scented and coloured flowers (*p* < 0.001) and those with wind simulation and unimodal coloured flowers (*p* < 0.001).

#### 3.2.3. Correct choices made after landing

The number of correct choices made after landing was different between the treatments and control (*χ*^2^_3_ = 26.14, *p* < 0.001, figure 4*c*). *Post hoc* comparisons revealed that foragers presented with wind and unimodal scented flowers made fewer correct choices after landing compared with foragers presented with no wind simulation and unimodal scented flowers (*p* = 0.002), those presented with wind and unimodal coloured flowers (*p* = 0.003) and those with wind and bimodal scented and coloured flowers (*p* < 0.0001).

### 3.3. Preference tests

Foragers had no innate preference to either peppermint or lavender scents (peppermint: 8.73 ± 2.74 (mean ± s.d.), lavender: 11.27 ± 2.74, *t*_14_ = 1.79, *p* = 0.095).

## 4. Discussion

We examined bumblebee foraging behaviour in the presence of one of two types of environmental noise which affect the transfer of volatiles: chemical interference and high wind speeds. Our results clearly show that both chemical interference and high wind speeds have detrimental effects on the foraging behaviour of *B. terrestris* in scented unimodal flowers, but the inclusion of an additional visual signal component to floral displays negated these effects. This suggests that in scenarios where olfactory communication is compromised, visual signal components can act as a backup, supporting the efficacy backup hypothesis as an explanation for the evolution and maintenance of multimodal floral signals.

Both chemical interference and high wind speeds caused foragers to take more flower visits to reach the learning criterion in unimodal scented flowers compared with foragers presented with unimodal scented flowers without either interference type (figures 3*a* and 4*b*). These results correspond with the claim by Wilson *et al.* [24] that olfactory communication between plants and pollinators is vulnerable to chemical interference and windy conditions. The total number of rewarding drinks as well as the number of correct choices made after landing was also lower in foragers presented with either of the two interference types and unimodal scented flowers compared with their counterparts which were not presented with interference (figures 3 and 4). These results complement a previous study [23] in which background odours affected the ability of hawkmoth *Manduca sexta* to correctly navigate an odour plume to its source. The detrimental effects on learning time, the total number of rewarding drinks and the number of correct choices made after landing were mitigated in foragers presented with bimodal floral displays and either interference type. The increase in post-landing accuracy also complements a previous study [17] where the converse possibility was explored and similar accuracy benefits were found when olfactory signals were used as a backup for visual signals in low-light conditions.

These detrimental effects to foraging efficiency are likely to have negative consequences to both plant and pollinator. Reductions in total number of rewarding drinks would limit the net energy gained by individual bees during foraging and subsequently the colony, negatively affecting survival, growth and reproductive output [46]. A decrease in correct choices made after landing may also be detrimental to plant fitness via increased clogging of stigmas by foreign pollen or pollen loss on interspecific species [47,48]. Our findings suggest that in windy habitats with crowded, scented vegetation (e.g. common to Mediterranean habitats), coloured flowers with species specific fragrances would enhance pollinator constancy and foraging efficiency [49,50]. This implies that visual stimuli can act as a backup for olfactory stimuli and that multimodal displays are more reliable stimuli in the presence of olfactory interference, giving further support to the efficacy backup hypothesis. These results mirror a previous study [17] in which olfactory stimuli were found to act as a backup for visual signals for flowers at different levels of illumination. In the light of the previous study [17], our own findings, and the observation of honeybees using scent as the primary discriminating factor over colour [39], it is possible that both visual and olfactory modalities backup each other, with bees using whichever stimuli are most conspicuous at the time. This also complements a previous study [51] in which bumblebees used spatial arrangements of either visual or olfactory stimuli to reduce nectar discovery times.

Adverse effects to forager learning times also suggest that both chemical and wind interference can affect associative learning in *B. terrestris*. This effect on learning may have detrimental effects to the level of flower constancy a forager reaches, lessening benefits to the plant, and potentially increase the costs associated with switching plant species for the pollinator [52,53]. This also suggests that flower constancy resulting from multimodal learning is more beneficial to flowers than unimodal learning in the presence of environmental variability. These findings are particularly relevant to nocturnal or crepuscular flowers and pollinators, such as hawkmoths, which rely heavily on olfactory cues to identify and discriminate between flowers, putting them at particular risk of noise which compromises olfactory communication [54–57]. Although nocturnal, these foragers can still incorporate visual display components into their search behaviour as seen in the hawkmoth *Deilephila elpenor*, which can use colour vision to discriminate between coloured stimuli in light condition equivalent to dim starlight [58].

Curiously, chemical interference also impeded foraging on purely visual artificial flowers (figure 3). This could be an indication of multisensory integration whereby information from one sensory modality influences processing in another modality [59], which has been discussed in relation to bee vision and olfaction [60]. This also implies that chemical interference may be detrimental to foraging efficiency even when visual cues are available. Alternatively, this effect of impeded foraging may also be caused by the essential oils overstimulating the odour receptor cells causing disorientation in the bees, which occurs with other volatiles such as naphthalene [61]. If this hindered foraging efficiency is caused by essential oil induced disorientation, our findings suggest that multimodal stimuli may mitigate the effects of insect repellents.

Considering the results, chemical interference has the potential to affect plant–animal interactions in multiple ways. Flowering plants and pollinators inhabiting environments with high plant species richness, such as tropical forests [62] or Mediterranean climatic regions [63], would be particularly susceptible to disruption by chemical interference. Chemical interference could also affect pollinator behaviour in environments with lower plant species richness if the perceptual systems of a pollinator experiences a similarity between odours [64]. Depending on the perceptual similarity of available odours, their concentration, and the variation of odours experienced during their learning, this phenomenon (referred to as olfactory generalization) could occur in areas of lower plant species richness [65].

Pollinators may mitigate these effects of olfactory noise through perceptual filtering, whereby only particular odours present in complex odour blends are detected by the antenna and only a select few of these detected volatiles elicit behavioural responses [66–69]. This perceptual filtering could assist in the location of particular flowers in the presence of multiple VOCs. On the other hand, olfactory noise could be beneficial to plants that are at risk of attracting herbivores through their volatiles [8], or to food-deceptive Batesian mimic flowers [70] which could potentially receive more visits from pollinators if the olfactory signals of rewarding flowers have compromised efficacy during learning or foraging.

These effects of chemical interference also relate to atmospheric pollution, which affects all terrestrial ecosystems and potentially affects VOC transfer at a global level [71]. McFrederick *et al.* [64] propose that the distance at which pollinators can detect highly reactive volatiles has changed from kilometres during pre-industrial times to less than 200 m in modern times due to the destruction of volatiles via chemical reactions with atmospheric pollutants. It would be of interest to know which VOCs are particularly reactive to atmospheric pollutants and consequently which plants, pollinators and environments are put at particular risk by these pollutants. Wilson *et al.* [22] suggest that characterizing entire ‘odoromes’, the collective scent profile of habitats, would be a useful tool in understanding the baseline levels of olfactory noise that insects encounter as well as increasing our understanding of how airborne pollutants from anthropogenic sources affect volatile signalling. With these data, we would also gain insights into which habitats have collective scent profiles that facilitate or hinder the transmission of volatile signals used by pollinators, herbivores or insect parasitoids and predators. It would also be valuable to explore other interactions which would be affected by chemical interference and turbulence caused by increased wind speeds in contexts other than pollination, as VOCs play important roles in other multitrophic interactions including the repelling of herbivores, attracting predators and parasitoids and signalling to other plants [72,73].

It is worth noting that in some instances, the inclusion of additional blends of floral volatiles may not contribute towards noise or decreases in the efficacy of a learned VOC blend. In cases where odour blends have no chemical overlap or have shared compounds that are not perceived by the forager, it is unlikely that there would be any detrimental effects to signal transmission. It has also been speculated that in some instances, the presence of additional and contrasting VOC blends may enhance a response to a learned VOC blend by highlighting the perceptual contrast [74]. However, the detrimental effects demonstrated in the treatments with chemical interference imply that this is not the case with the VOCs used within our study.

In terms of interference through high wind speeds, we saw detrimental effects to learning speeds, nectar collection rate and post-landing accuracy, mirroring the negative effects found in previous studies [35,37]. This disruption to foraging behaviour is likely to be caused by turbulent air movements stretching, compressing and tearing apart odour filaments alongside the creation of odour-free gaps, creating difficulties when locating the source of the odour [20,75]. It is also unknown if the flight arenas used within the experiments are large enough to allow for this formation and subsequent degradation of odour plumes which are of ecologically relevant sizes. Therefore, field experiments done in natural wind and airborne chemical environments of study organisms would be beneficial. Alpine meadows [76] and Mediterranean climactic regions [77] would make appropriate study locations as habitats at risk of this wind-related noise. Wind speeds are also projected to increase in certain environments due to climate change [78,79]. Depending on the environment, these increases in wind speed may possibly decrease the distance at which plants can elicit a behavioural response from their pollinators through odour plume disruption [80].

Communication between plants and their visitors is of extreme importance to all terrestrial ecosystems and by understanding the factors which influence the evolution and effectiveness of this information transfer we gain a great insight into how this relationship can be affected in a changing world. Our results suggest that both chemical interference and high wind speeds have negative effects on the foraging behaviour of bumblebees *B. terrestris* in scented unimodal flowers, but the inclusion of an additional visual signal component to the floral displays negated these negative effects. This suggests that visual signal components can act as a backup in environments where olfactory communication is compromised, benefitting both plant and pollinator, and supporting the efficacy backup hypothesis as an explanation for the evolution and maintenance of multimodal floral signals. It is our hope that this study provides a proof of concept for these effects on the transmission of olfactory signals and inspires future research into which areas these interference types are most (or least) likely to occur, and at what spatial scales.

## Supplementary Material

Supplementary materials - Tables

## Supplementary Material

Supplementary materials - Data

## Data accessibility

The datasets supporting this article have been uploaded as part of the supplementary material. Data are available from the Dryad Digital Repository, http://dx.doi.org/10.5061/dryad.3g591 [81].
