# Supplementary material for: Colour as a backup for scent in the presence of olfactory noise: testing the efficacy backup hypothesis using bumblebees (Bombus terrestris)
Source: R Soc Open Sci. 2017 Nov 29;4(11):170996. doi: 10.1098/rsos.170996 (PMC5717666; doi:10.1098/rsos.170996)
Supplement: Supplementary materials - Tables [file rsos170996supp1.pdf]

Supplementary Materials

Table S1. Experimental components of conditions used within the chemical interference experiment detailing the presence or absence of visual or scent flower components as well as the presence of chemical interference.

Group	Condition	Scented Flowers	Chemical interference	Visual Cue	*n*
A	No chemical interference and unimodal scented flowers	✓	✗	✗	18 (Colony C: 6, Colony D: 12)
B	With chemical interference and unimodal scented flowers	✓	✓	✗	18 (Colony C:4, Colony D: 14)
C	With chemical interference and bimodal scented and visual flowers	✓	✓	✓	18 (Colony D: 18)
D	With chemical interference and unimodal visual flowers	✗	✓	✓	18 (Colony CCC: 4, Colony DD: 8, Colony F: 1, Colony: E 4, Colony CCC: 1)

Table S2. Experimental components of conditions used within the wind simulation experiment detailing the presence or absence of visual or scent flower components as well as the presence of simulated wind.

Group	Condition	Scented Flowers	Air Movement	Visual Cue	*n*
A	No air movement and unimodal scented flowers	✓	✗	✗	18 (Colony A: 7, Colony F: 11)
B	With air movement and unimodal scented flowers	✓	✓	✗	18 (Colony F: 10, Colony D: 8)
C	With air movement and bimodal scented and visual flowers	✓	✓	✓	18 (Colony A: 10, Colony F: 8)
D	With air movement and unimodal visual flowers	✗	✓	✓	18 (Colony EE: 12, Colony FF: 6)
